# A survey of clinical and laboratory characteristics of the dengue fever epidemic from 2017 to 2019 in Zhejiang, China

**DOI:** 10.1097/MD.0000000000031143

**Published:** 2022-10-21

**Authors:** Ze-Ze Ren, Yi Zheng, Tao Sun, Gang-Yi Wang, Xiao-Mei Chen, Yu-Mei Zhou

**Affiliations:** a Department of Infectious Disease, The Second Affiliated Hospital of Zhejiang Chinese Medicine University, Hangzhou, Zhejiang, China.

**Keywords:** clinical features, dengue fever, epidemiology, immune cells, laboratory characteristics

## Abstract

To explore the epidemic, clinical, and laboratory characteristics of dengue patients in Zhejiang and the possible mechanism. Epidemic, clinical and laboratory data of 231 dengue patients admitted to the Second Affiliated Hospital of Zhejiang Traditional Chinese Medicine University between August 2017 and December 2019 were collected. GSE43777 dataset was downloaded from the Gene Expression Omnibus database and was used for the immune cell infiltration analysis, logistic regression analysis, and nomogram construction. Gene set enrichment analysis (GSEA) was performed to explore the possible regulatory pathways in dengue infection. Further, the receiver operating characteristic curve analysis and decision curve analysis were conducted to evaluate the value of related immune cells in predicting dengue severity. Among the 231 patients, the gender ratio was 1:1.1 (male/female). The patients in the <60 years age group, 60 to 80 years age group, and >80 years age group were 47.2%, 45.5%, and 7.3%, respectively. The major symptoms were fever (100%), weak (98.3%), anorexia (76.6%), muscle and joint pain (62.3%), and nausea (46.8%). In dengue patients, 98.7% of serum samples had decreased platelet levels, 96.5% of them had decreased white blood cell (WBC) levels, 97.8% had elevated aspartate aminotransferase levels, 82.3% had elevated lactate dehydrogenase levels, 49.4% had increased creatinine levels, and 35.5% had increased creatine kinase levels. Pneumonia, pleural effusion, and bilateral pleural reaction were observed in 16.5%, 8.2%, and 4.8%, respectively of dengue patients. Gallbladder wall roughness and splenomegaly accounted for 6.1% and 4.3% of all cases. Moreover, the levels of T cell, B cell, and dendritic cells were significantly higher in the convalescent group and they were involved in immune- and metabolism-related pathways. Of note, low levels of these 3 immune cells correlated with high dengue infection risk, while only dendritic cells exhibited satisfactory performance in predicting dengue severity. Dengue fever patients often onset with fever, accompanied by mild abnormalities of the blood system and other organ functions. Moreover, T cells, B cells, and dendritic cells might be involved in dengue infection and development.

## 1. Introduction

Dengue is the most prevalent emerging arthropod-borne viral infection worldwide, secondary only to malaria in terms of morbidity and mortality.^[1]^ Transmitted by the female Aedes mosquitos, dengue has rapidly spread in the past few decades, which has been further driven by economic booms, unprecedented international travel, and climate change.^[2–5]^ The rapid increase in dengue incidence has become a serious public health threat, especially in Southern Africa, Southeast Asia, and the Western Pacific Ocean regions.^[6,7]^ Approximately 4 billion people are at risk of contracting dengue and 390 million dengue infections occur annually, of which 96 million are recognized clinically including 20,000 deaths.^[8]^ Dengue virus (DENV) belongs to the family Flaviviridae and is a single-stranded, positive-sense, ribonucleic acid (RNA) virus with a genome of 10.7 kb.^[9]^ Infection by any 1 of 4 antigenically distinct serotypes (DENV1-4) can lead to various degrees of disease symptoms ranging from nonspecific febrile illness to classic dengue fever, dengue hemorrhagic fever, and dengue shock syndrome.^[10]^ Currently, a combination of clinical dengue symptoms, virus isolation, viral RNA detection, and serological assays are available for the diagnosis of dengue fever; however, none of them are sufficiently sensitive and specific to be used as a stand-alone diagnostic tool.^[11]^

The clinical outcomes of infection depend on a complex interaction between DENV and the host immune response. While the protective immune response is required for viral clearance, the detrimental immune reaction is the major cause of plasma leakage, cytokine storm, viral propagation, and severe dengue hemorrhagic fever/dengue shock syndrome.^[12]^ Innate and innate-like responses to DENV infection are not only critical as a first line of defense but also influence subsequent adaptive T and B cell responses.^[13]^ In addition to the role of innate-like T cells, and natural killer T cells, multiple innate responses play a vital role in DENV infection and viral invasion strategies, particularly dendritic cells, macrophages, and monocytes.^[14–17]^ Therefore, further exploration of the role of immune cells in dengue is urgently required to provide other possible therapeutic strategies.

This study aims to retrospectively investigate the epidemiological features, clinical manifestations, and laboratory characteristics of dengue. Moreover, we performed efficient bioinformatics and statistical analyses to explore the potential roles of immune cells and regulatory pathways in dengue.

## 2. Materials and Methods

### 2.1. Clinical samples

Dengue patients admitted to the Second Affiliated Hospital of Zhejiang Traditional Chinese Medicine University between August 2017 and December 2019 were included in this retrospective study. Patient electronic medical records were reviewed for epidemiological characteristics, clinical features, laboratory findings, and imaging examinations. All subjects who were contacted by telephone and agreed to participate gave verbal informed consent. The study was approved by the ethics committee of our hospital (approval number: 2021-LW-009-01).

### 2.2. Diagnostic criteria

Clinical diagnosis of dengue was made according to the Guideline on Diagnosis and Therapy of Dengue Fever (Edition 2, 2014), issued by the National Health and Family Planning Commission of the People’s Republic of China.^[18]^ The diagnostic criteria were as follows: with the clinical manifestations of dengue such as fever, headache, muscle or joint pain, rash, bleeding tendency; with epidemiological history (had traveled to a dengue-endemic area within 15 days prior to the onset of illness, or there was a case of dengue in his/her residence; decreased white blood cell (WBC) or platelet (PLT) counts and positive serum DENV IgM antibodies. Laboratory confirmed cases: clinically diagnosed cases with dengue NS1 antigen detected in serum, DENV isolated or convalescent serum with a more than 4-fold increase in specific IgG antibody titer.

### 2.3. Examinations

Blood counts were completed by an automatic blood cell analyzer (SYSMEX XE-5000) for all patients. Detection Kit analyzed laboratory diagnostic tests including DENV-RNA, DENV-IgM/IgG, and NS1 antigen detection for DENV RNA (RT-PCR-Fluorescence Probing, Hua yin Medical Technology Co Ltd, Guangzhou, China), Diagnostic Kit for Dengue IgG/IgM Antibody (Colloidal Gold, Wondfo Biotech, Guangzhou, China) and Diagnostic Kit for Dengue VirusNS1 (ELISA, WANTAI BioPharm, Beijing, China). Laboratory Biochemical tests were analyzed by Cobas 8000 (Roche Diagnostics GmbH). Pneumonia and hydrothorax were assessed by computed tomography. Ultrasonic testing was performed to observe liver and gall.

### 2.4. Bioinformatics analyses

Since the host-specific immune responses to DENV likely represent an essential role in the pathophysiology of the disease and subsequent clinical manifestation of dengue infection,^[13]^ immune cell infiltration analysis was performed to explore the possible mechanism of dengue. Gene Expression Omnibus database (https://www.ncbi.nlm.nih.gov/geo/) was used to obtain the dengue microarray dataset by setting the following filter: more than 50 samples with febrile and convalescent information; with expression profiling data; with dengue severity information. Finally, the GSE43777 dataset containing 269 samples was chosen for the following analyses.

The microenvironment cell populations-counter algorithm was employed to estimate the 8 immune cells and 2 stromal cells’ infiltration levels in patients in the febrile phase and convalescent phase. Further, the gene set enrichment analysis (GSEA) was performed to explore the underlying mechanism of dengue. The gene set was permutated 1000 times. A nominal *P* value <.05 and a false discovery rate q-value < 0.25 were considered to be statistically significant.

### 2.5. Statistical analysis

Statistical analyses were performed using SPSS software (version 23.0, SPSS, Chicago) and R software (version 4.1.1). The Kolmogorov-Smirnov test was adopted to evaluate the normal distribution of the measurement data. The measurement data conforming to the normal distribution were represented as mean ± SD, and non-normally distributed measurement data were expressed as the median and quartile [M (P25, P75)]. The count data were expressed as numbers (percentages). One-sample *t* test was used to compare the means to the reference value.

The differential expression levels of immune cells and their gene markers between febrile and convalescent groups were analyzed using Student *t* test. The association of immune cells with dengue infection and severity was assessed by univariate logistic regression analysis. Based on the logistic regression analysis results, a nomogram was generated to determine which risk factor contributed most to dengue infection. The calibration curve was drawn to evaluate the accuracy of the nomogram. In addition, the receiver operating characteristic curve and area under the curve were used to assess the value of immune cells in predicting dengue severity. Decision curve analysis was performed to examine the potential effectiveness and benefits of the immune cells in dengue severity. *P* < .05 was considered statistically significant.

## 3. Results

### 3.1. Patient epidemiological characteristics

A total of 231 patients with dengue were included in the study including 110 males (47.6%) and 121 females (52.4%). The mean age of the patients was 57.7 ± 16.5 years (range: 16-91 years). Totally 213 cases met clinical diagnosis and 18 cases were laboratory confirmed. The patients in the < 60 years age group, 60 to 80 years age group, and >80 years age group were 47.2%, 45.5%, and 7.3%, respectively. The main onset peak appeared in September (63.7%) and August (35.5%) (Table 1).

**Table 1:** Epidemiological characteristics of the patients with dengue fever (n = 231).

Characteristics	Number of cases (n)	Percentage (%)
Gender		
Male	110	47.6
Female	121	52.4
Age (yrs)		
< 60	109	47.2
60-80	105	45.5
>80	17	7.3
Time of dengue onset		
August	82	35.5
September	147	63.7
October	1	0.4
November	1	0.4

### 3.2. Basic disease

There were 27 cases with fatty liver and 72 cases having hypertension. Patients with diabetes, coronary heart disease, arrhythmia, and cerebral infarction were 26, 8, 5, and 3, respectively. Chronic nephrosis was observed in 3 cases and asthma in 2 cases.

### 3.3. Clinical symptoms

The most frequent clinical manifestations were fever (100.0%), weak (98.3%), anorexia (76.6%), muscle or joint pain (62.3%), nausea or vomiting (46.8%), chills (45.0%), and dizziness (43.1%). Headache was observed in 39.8% of dengue patients. Both rash and diarrhea accounted for 22.5% of patients. 14.7% of patients had cough and expectoration. Abdominal pain (4.8%), chest discomfort (3.9%), sore throat (2.2%), gingival bleeding (1.7%), conjunctival hemorrhage (1.3%), epistaxis (0.9%), and chest pain (0.5%) occurred in a small proportion of patients (Table 2). Of note, all the 231 patients had different degrees of fever, and the average temperature was 38.9°C ± 0.6°C (range: 37.5°C-40.7°C). Besides, 52 patients developed rashes, which first appeared as early as the first day and as late as day 10 of the course of the disease, with an average of (5 ± 2) days and an average duration of (4 ± 2) days. Among them, 31 cases (13.4%) had congestive rashes and 21 cases (9.1%) had hemorrhagic rashes, mostly distributed in the abdomen, back, and limbs, especially in the lower extremities. The rash was mainly characterized by needle-like hemorrhagic spots.

**Table 2:** Clinical observations of the patients with dengue fever (n = 231).

Characteristics		Number of cases (n)	Percentage (%)
Fever	Yes	231	100.0
	No	0	0.0
Weak	Yes	227	98.3
	No	4	1.7
Anorexia	Yes	177	76.6
	No	54	23.4
Muscle or joint pain	Yes	144	62.3
	No	87	37.7
Nausea or vomiting	Yes	108	46.8
	No	123	53.2
Chills	Yes	104	45.0
	No	127	55.0
Dizziness	Yes	95	43.1
	No	136	58.9
Headache	Yes	92	39.8
	No	139	60.2
Rash	Yes	52	22.5
	No	177	76.6
	Unknown	2	0.9
Diarrhea	Yes	52	22.5
	No	179	77.5
Cough and expectoration	Yes	34	14.7
	No	197	85.3
Abdominal pain	Yes	11	4.8
	No	220	95.2
Chest discomfort	Yes	9	3.9
	No	222	96.1
Sore throat	Yes	5	2.2
	No	226	97.8
Bleeding gums	Yes	4	1.7
	No	227	98.3
Conjunctival hemorrhage	Yes	3	1.3
	No	110	47.6
	Unknown	118	51.1
Epistaxis	Yes	2	0.9
	No	229	99.1
Chest pain	Yes	1	0.5
	No	144	62.3
	Unknown	86	37.2

### 3.4. Laboratory examinations

Of the 231 patients, 228 patients (98.7%) had decreased PLT counts and 223 (96.5%) had decreased WBC counts. Elevated levels of c-reaction protein and thrombocytocrit were detected in about 47.6% and 46.8% of patients, respectively. Coagulation function is mainly characterized by the prolonged activated partial thromboplastin time occurring in 126 patients (54.5%), followed by lower fibrinogen in 51 cases (22.1%). Compared with the reference range, aspartate aminotransferase levels, alanine aminotransferase levels, and gamma-glutamyl transpeptidase levels were significantly higher in the dengue patients (all *P* < .001). 114 (49.4%) of 231 cases had higher creatinine levels, 190 (82.3%) of 231 cases had increased lactate dehydrogenase levels, and 82 (35.5%) of 231 cases had upregulated creatine kinase levels. There were 54 cases of positive urinary protein, 49 cases of positive urinary occult blood, and 57 cases of positive fecal occult blood (Table 3).

**Table 3:** Laboratory examinations of the patients with dengue fever (n = 231).

Characteristics	Number of cases (n)	Percentage (%)	M (*P*_25_, *P*_75_)	Reference range
PLT (*10^9/L) ↓	228	98.7	41 (26, 63)***	125-350
WBC (*10^9/L) ↓	223	96.5	1.8 (1.5, 2.2) ***	4.0-10.0
LDH (U/L)↑	190	82.3	341 (291, 414) ***	120-250
CRP (mg/L) ↑	110	47.6	22 (16, 29) *	0-10
PCT (ng/mL) ↑	108	46.8	0.13 (0.09, 0.20)	0-0.05
APTT (s) ↑	126	54.5	42 (38, 46) ***	22-36
FIB (g/L)↓	51	22.1	1.83 (1.71, 1.90) **	2.0-4.0
PT (s) ↑	18	7.8	14.4 (13.7, 16.4) ***	10.0-13.5
AST (U/L) ↑	226	97.8	110.0 (75.0, 170.5) ***	13-40
ALT (U/L) ↑	164	71.0	94.0 (64.3, 138.8) ***	7-50
CR (μmol/L) ↑	114	49.4	112.4 (92.1, 119.9) ***	41.0-111.0
CK (U/L) ↑	82	35.5	391.5 (279.5, 759.75)	40-310
GGT (U/L) ↑	77	33.3	90.0 (69.5, 149.5) ***	7-60
Stool occult blood (+)	57	24.7		
Urine protein (+)	54	23.4		
Urinary occult blood (+)	49	21.2		

↓ indicates the results below the lower limit of the reference range;

↑ indicates the results with a higher upper limit of the reference range. Compared with the lower limit of the reference range or the higher upper limit of the reference range,

* *P* < .05,

** *P* < .01,

*** *P* < .001.

ALT = alanine aminotransferase, APTT = activated partial thromboplastin time, AST = aspartate aminotransferase, CK = creatine kinase, CR = creatinine, CRP = c-reaction protein, FIB = fibrinogen, GGT = gamma-glutamyl transpeptidase, LDH = lactate dehydrogenase, PCT = thrombocytocrit.

### 3.5. Imaging examinations

Chest computed tomography examination showed that 38 cases had pneumonia and a small pleural effusion existed in 19 cases. Bilateral pleural reaction occurred in 11 cases (4.8%), increased lung texture and coarseness of lung texture in 5 cases (2.2%), and bilateral pleural thickening in 4 cases (1.7%). Ultrasonic examination exhibited that gallbladder wall roughness occurred in 14 cases (6.1%), splenomegaly in 10 cases (4.3%), gallbladder wall edema in 3 cases (1.3%), and a small amount of pericardial effusion in 3 cases (1.3%).

### 3.6. Immune cell infiltration analysis

**Figure 1.**
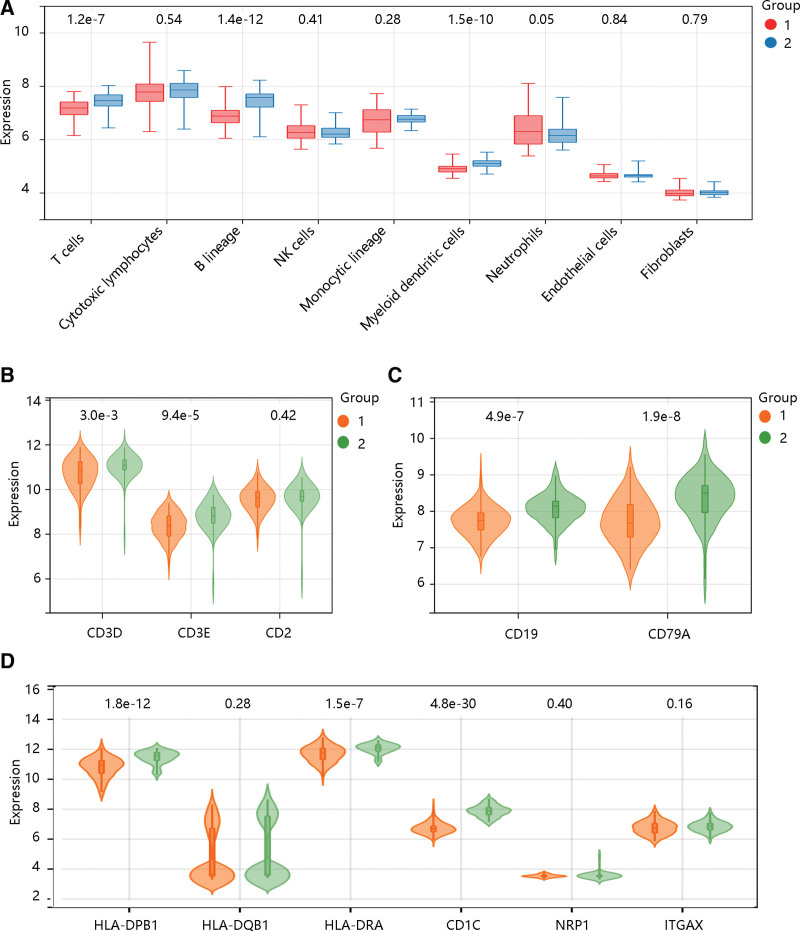
The immune cell infiltration analysis in dengue using MCP-counter algorithm. **(A**) Several immune cell infiltration levels in febrile and convalescent groups. The expression levels of gene markers of **(B**) T cells, **(C**) B lineage, and **(D**) Dendritic cells in febrile and convalescent groups. Group: 1, febrile; 2, convalescent. MCP = microenvironment cell populations.

To explore the underlying mechanism of dengue, immune cell infiltration analysis was firstly conducted using the microenvironment cell populations-counter algorithm. The result showed that T cells, B lineage, and myeloid dendritic cells were significantly higher in the convalescent group (all *P* < .001) (Fig. 1A). Then, we analyzed the expression of marker genes of T cells, B lineage, and myeloid dendritic cells in the febrile and convalescent groups. The notable expression levels of CD3D, CD3E, CD19, CD79A, HLA-DPB1, HLA-DRA, and CD1A were observed in the convalescent group (all *P* < .01) (Fig. 1B–D).

### 3.7. T cells, B cells, and dendritic cells predicted dengue infection

**Figure 2.**
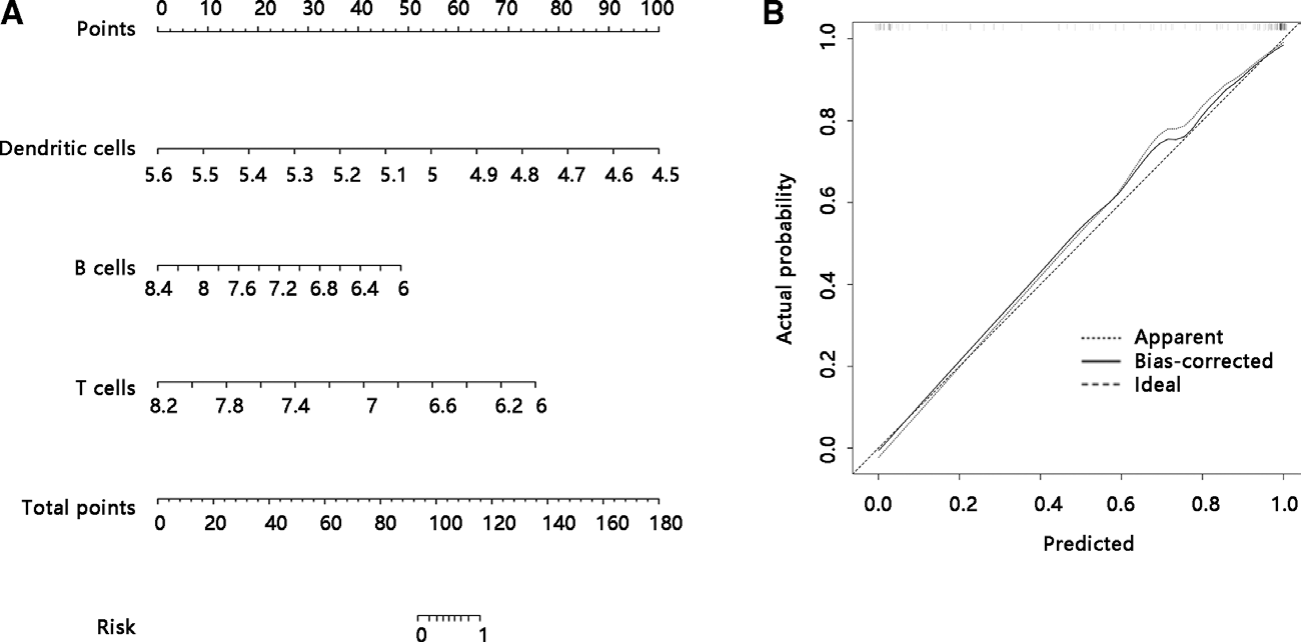
A nomogram for predicting the risk of dengue infection. **(A**) The dengue risk model was developed using T cells, B cells, and dendritic cells. **(B**) The calibration curve of the nomogram for dengue infection risk.

To evaluate the predictive value of T cells, B cells, and dendritic cells for dengue infection risk, we performed univariate logistic regression analysis, and found that low expression levels of T cells, B cells, and dendritic cells were significantly related to high dengue infection risk (all *P* < .001) (Table 4). Following this, these 3 immune cells were included into nomogram generation and dendritic cells contributed most to predict the risk of dengue infection, followed by T cells and B cells (Fig. 2A). The calibration curve of the nomogram to predict the dengue infection risk showed good agreement (Fig. 2B). These findings suggested that T cells, B lineage, and myeloid dendritic cells might occupy a crucial role in dengue infection.

**Table 4:** The association of immune cells with dengue infection using univariate logistic regression analysis.

Immune cells	Odds ratio	95% confidence interval	*P* value
T cells	0.053	0.014-0.193	<.001
B cells	0.025	0.008-0.081	<.001
Dendritic cells	0.004	0.003-0.007	<.001

### 3.8. GSEA

**Figure 3.**
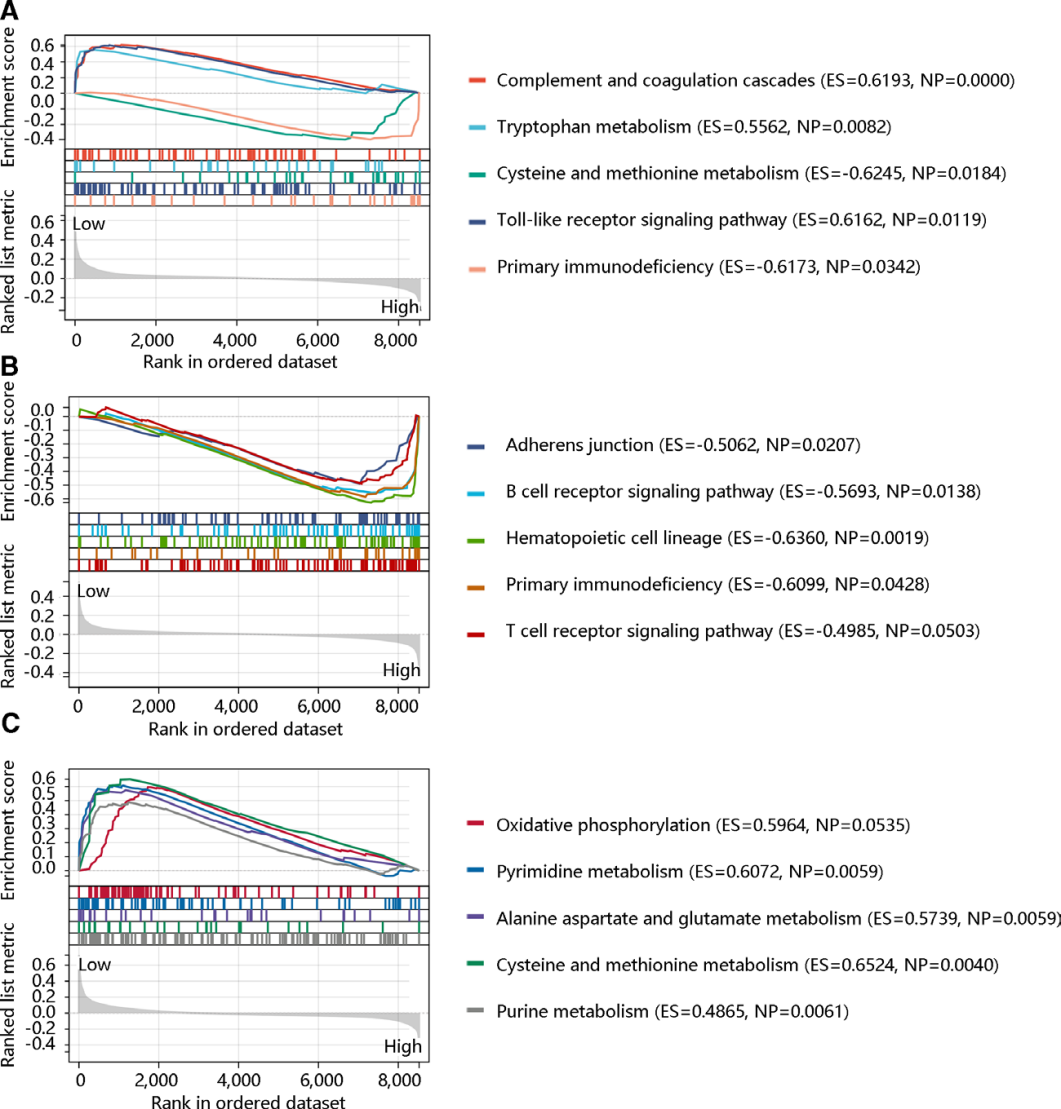
Gene set enrichment analysis. **(A**) The top pathways enriched in the high-T cell level group and low-T cell level group. **(B**) The top 5 pathways enriched in the high-B lineage level group. **(C**) The main pathways enriched in the low-dendritic cell level group.

To investigate the pathological role of T cells, B cells, and dendritic cells in dengue infection, patients were divided into 2 groups by setting the median level of T cells, B lineage, and myeloid dendritic cells as the threshold for GSEA. As shown in Figure 3A, complement and coagulation cascades, tryptophan metabolism, and cysteine and methionine metabolism were enriched in the low-T cell level group. Toll-like receptor signaling pathway and primary immunodeficiency were enriched in the high-T cell group. Adherens junction, B cell receptor signaling pathway, hematopoietic cell lineage, primary immunodeficiency, and T cell receptor signaling pathway were mainly enriched in the high-B lineage level group (Fig. 3B). However, oxidative phosphorylation, pyrimidine metabolism, alanine aspartate, glutamate metabolism, cysteine and methionine metabolism, and purine metabolism were the major pathways in the low-dendritic cell level group (Fig. 3C). These results revealed that T cells, B lineage, and myeloid dendritic cells might participate in the dengue infection through immune and metabolism-related pathways.

### 3.9. Dendritic cells predicted dengue severity

**Figure 4.**
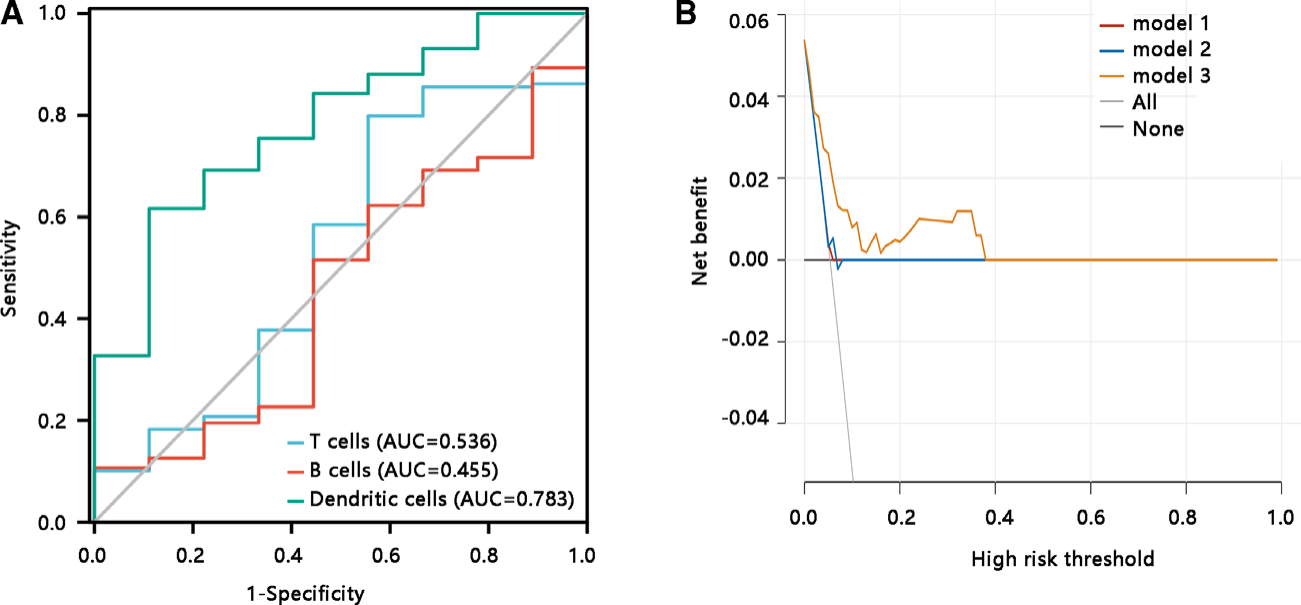
Dendritic cells showing predictive power for dengue severity. **(A**) Receiver operating characteristic curve of the T cells, B cells, and dendritic cells for dengue severity. AUC, area under the curve. **(B**) Decision curve analysis of the T cells, B cells, and dendritic cells for dengue severity. Model 1: T cells; model 2: B cells; model 3: dendritic cells.

After characterizing the essential role of T cells, B cells, and dendritic cells in dengue infection, we further explored the value of these 3 immune cells in predicting dengue severity. Univariate logistic regression analysis showed that T cells and B cells levels had no remarkable relationship with dengue severity (all *P* > .05), but low level of dendritic cells was correlated with dengue severity with an odds ratio of 0.012 (*P* < .05) (Table 5). Further, receiver operating characteristic and decision curve analysis analyses were performed to compare the efficacy of the 3 immune cells in predicting dengue severity. Dendritic cells performed well in distinguishing dengue fever from dengue hemorrhagic fever compared with T cells and B cells. The area under the curves for T cells, B cells, and dendritic cells were 0.536, 0.455, and 0.738, respectively (Fig. 4A). Besides, dendritic cells had highest clinical net benefit (Fig. 4B). The above findings revealed that dendritic cells exhibited superior ability in predicting dengue severity.

**Table 5:** The association of immune cells with dengue severity using univariate logistic regression analysis.

Immune cells	Odds ratio	95% confidence interval	*P* value
T cells	0.380	0.045-3.196	.373
B cells	2.769	0.325-23.601	.351
Dendritic cells	0.012	0.001-0.163	.014

## 4. Discussion

As a mosquito-borne infectious disease with the fastest transmission speed in the world, dengue fever has become 1 of the diseases threatening global public health.^[19]^ The influence of climate change and travel to/from endemic countries are important factors leading to the disease outbreak.^[20]^ An analysis of imported cases from 2014 to 2018 in China showed that the dengue patients were mainly aged between 21 and 50 years old made up the most farmers and unemployed people.^[21]^ Among the 231 dengue patients admitted to our hospital, those in the < 60 years age group accounted for the largest proportion, followed by the 60 to 80 years age group, which may be attributed to the fact that more elderly patients come to see a doctor. The onset time was concentrated in August and September every year, which was consistent with the peak time of case reports in Pan’s study.^[22]^ In the south of China, an increase in temperature and humidity during the summer favors the growth of mosquitoes breeding and dengue fever.

The cellular immune response contributes to the clinical manifestations of dengue. Production of the pyrogen IL-1β begins with the first wave of infected cells and TNF began at the stage of skin infection responsible for fever, muscle pain, and anorexia.^[23]^ In addition, type 1 interferon contributes to various clinical symptoms, the range of which is clearly delineated during the administration of exogenous interferon in a therapeutic setting: fatigue, headache, chills, and fever.^[24]^ In this study, there were 229 cases of dengue fever and 2 cases of severe dengue fever. Most of the patients had acute onset and the main clinical manifestations were fever, fatigue, anorexia, muscle and joint pain, nausea, etc, which was generally consistent with the results of previous literature.^[1]^

Decreased WBC and/or PLT is 1 of the necessary conditions for the diagnosis of dengue fever. 96.5% of cases had decreased WBC, 98.7% of cases had decreased PLT, and some patients had mild abnormalities in coagulation indicators such as prolonged prothrombin time, and declined FIR, which lead to bleeding in the conjunctiva, nasal mucosa, and gingiva.^[25]^ The reduction of WBC and PLT may be due to the inhibitory effect of the DENV on hematopoietic stem cells and megakaryocytes in bone marrow.^[26]^ The DENV also causes damage to the body’s vital organs. If other causes of co-infection or liver damage are excluded, severe dengue patients are more likely to have abnormal alanine aminotransferase and aspartate aminotransferase levels.^[27]^ 114 cases (49.4%) of dengue patients had an abnormal renal function, mainly with mild elevation of creatinine, which was transient. Old age, obesity, diabetes history, and severe dengue fever combined with bacterial infection are related risk factors for acute kidney injury, leading to poor renal prognosis, longer hospital stay, and even higher mortality in dengue patients.^[28,29]^ DENV infection is characterized by cardiac involvement. In this study, 82 (35.5%) had a transient increase of creatine kinase, 3 (1.3%) had a small amount of pericardial effusion and occasionally had chest tightness and pain. Direct virus invasion, immune mechanism, electrolyte imbalance, intracellular calcium storage disturbances, lactic acidosis, and ischemia due to hypotension play an essential role in myocardial dysfunction.^[30]^ In terms of auxiliary examination, imaging findings in dengue are nonspecific, but they may assist in identifying severe dengue cases. The most common imaging findings in dengue are bilateral areas of ground-glass opacity or consolidation and bilateral pleural effusions.^[31]^ In addition, the thickening of the gallbladder wall by ultrasonography is considered a reliable basis for the rapid identification of potentially severe dengue cases.^[32]^

**Figure 5.**
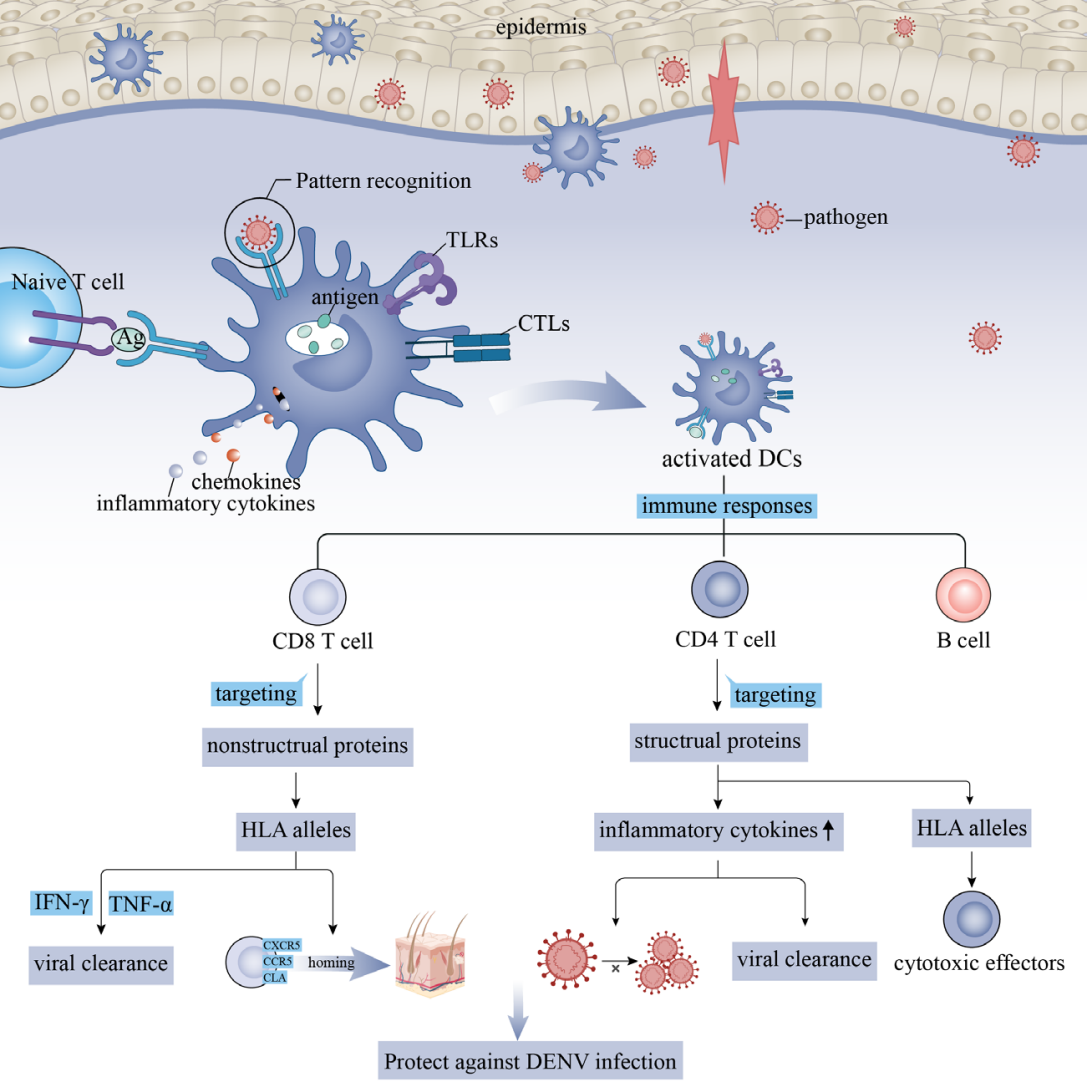
The diagram about the possible mechanism of T cells, B cells, and dendritic cells involved in dengue infection.

Following this, we investigated the underlying mechanism of dengue by immune infiltration analysis. The imbalanced and deregulated cell-mediated immunity is a pivotal component.^[33]^ Dendritic cells reside and migrate into barrier tissues such as the skin and mucosal epithelium that are the sites of pathogen invasion. Dendritic cells exhibit high levels of phagocytic activity, take up antigens, and detect pathogens through pattern recognition receptors. Additionally, dendritic cells express C-type lectins and Toll-like receptors as transmembrane proteins as well as intracellular sensors including cGAS, MDA-5, and RIG-I that recognize conserved microbial patterns.^[34,35]^ Upon pathogen, dendritic cells are activated, producing inflammatory cytokines and chemokines and presenting antigen to prime naïve T cells.^[36]^ Besides, dendritic cells line innate and adaptive immune responses by integrating innate signals from pathogen-associated microbial patterns with pathogen-derived antigens to induce antigen-specific T cell and B cell responses.^[17]^ The CD8 T cell response to DENV mainly targets to the nonstructural proteins NS3, NS4B, and NS5.^[37]^ In the populations previously exposed to DENV, the magnitude and breadth of CD8 T cell response correlated with HLA alleles associated with disease susceptibility, indicating that high magnitude/breadth of CD8 T cell response is linked to protection.^[38]^ DENV-specific CD8 T cells produce IFN-γ and TNF-α and the majority of these cells were effector memory T cells. Besides, DENV-specific CD8 T cells also express CXCR5, CCR5, and CLA; these cells can home to skin during dengue infection.^[38,39]^ The anti-DENC CD4 T cell response targets structural proteins.^[40]^ The CD4 T cells produce inflammatory cytokines, facilitating control of DENV replication and mediating viral clearance. In addition, CD4 T cells that expand depending on HLA alleles are likely cytotoxic effectors. Taken together, CD8 T cells and CD4 T cells may protect against DENV infection due to their association with HLA alleles and cytotoxic effector functions.^[41,42]^ The possible mechanism of dendritic cells, T cells, and B cells involved in dengue infection was shown in Figure 5, which requires further validation in the future. Our study found that high levels of T cell, B cell, and dendritic cell in the convalescent group than those in the febrile group. The low expression levels of these 3 immune cells correlated to high dengue infection risk. The authors speculated that dendritic cells, T cells, and B cells might play an important role in priming protective immune responses against human pathogens, and the lack of these immune cells might contribute to the pathogenesis and development of dengue. The GSEA result revealed that T cells, B cells, and dendritic cells were involved in immune- and metabolism-related pathways such as complement and coagulation cascades, Toll-like receptor pathway, B cell receptor signaling pathway, hematopoietic cell lineage, primary immunodeficiency, and purine metabolism pathway. Therefore, the 3 immune cells might affect the dengue infection through activation of these pathways. For further exploration, we found that dendritic cells presented superior ability in predicting dengue severity compared with T cells and B cells. However, the underlying mechanism still needs further investigation.

In conclusion, dengue fever patients often onset with fever, accompanied by mild abnormalities of the blood system and other organ functions. For confirmed patients, potential severe dengue cases should be identified in time, and active and perfect treatment should be given to reduce the mortality rate. Moreover, T cells, B cells, and dendritic cells might affect the dengue infection and development via immune- and metabolism-associate pathways.

## Author contributions

**Conceptualization:** Ze-Ze Ren.

**Data curation:** Ze-Ze Ren, Yu-Mei Zhou.

**Formal analysis:** Yi Zheng, Gang-Yi Wang.

**Investigation:** Yi Zheng.

**Methodology:** Yi Zheng.

**Resources:** Tao Sun, Yu-Mei Zhou.

**Supervision:** Tao Sun, Xiao-Mei Chen.

**Validation:** Tao Sun, Xiao-Mei Chen.

**Visualization:** Gang-Yi Wang, Xiao-Mei Chen.

**Writing – original draft:** Ze-Ze Ren, Tao Sun, Gang-Yi Wang, Xiao-Mei Chen, Yu-Mei Zhou.

**Writing – review & editing:** Gang-Yi Wang, Yu-Mei Zhou.
